# Inactivation of *E. coli* Using Atmospheric Pressure Plasma Jet with Dry and Wet Argon Discharges

**DOI:** 10.3390/membranes11010046

**Published:** 2021-01-09

**Authors:** Atif H. Asghar, Omar B. Ahmed, Ahmed Rida Galaly

**Affiliations:** 1 Department of Environmental and Health Research, The Custodian of the Two Holy Mosques Institute for Hajj and Umrah Research, Umm Al-Qura University, Makkah 24381, Saudi Arabia; ahasghar@uqu.edu.sa (A.H.A.); obmohamed@uqu.edu.sa (O.B.A.); 2 Department of Engineering Science, Faculty of Community, Umm Al-Qura University, Makkah 24381, Saudi Arabia; 3 Department of Physics, Faculty of Science, Beni-Suef University, Beni-Suef 62521, Egypt

**Keywords:** non-thermal APPJ, dry and wet Ar discharges, inactivation of *E. coli*, direct and indirect exposure, magnetic field, membrane damage mechanism

## Abstract

The acceleration of inactivating viable cells of Escherichia coli (*E. coli*), by using new direct and indirect innovative methods, is the targeted method of using an atmospheric pressure plasma jet (APPJ) operated by an AC high-voltage power source with variable frequency up to 60 kHz and voltage ranging from 2.5 to 25 kV. Discharges using dry argon (0% O_2_) discharges and different wet argon discharges using admixtures with O_2_/Ar ratios ranging from 0.25% to 1.5% were studied. The combined effects of dry and wet argon discharges, direct and indirect exposure using a mesh controller, and hollow magnets were studied to reach a complete bacterial inactivation in short application times. Survival curves showed that the inactivation rate increased as the wettability increased. The application of magnetized non-thermal plasma discharge with a 1.5% wetness ratio causes a fast inactivation rate of microbes on surfaces, and a dramatic decrease of the residual survival of the bacterial ratio due to an increase in the jet width and the enhanced ability of fast transport of the charges to viable cells, especially at the edge of the Petri dish. The membrane damage of *E. coli* mechanism factors in the activation process by APPJ is discussed.

## 1. Introduction

One of the fashionable targets in our life is to attain an eco-city and maintain good health. Therefore, plasma plays an important role in the inactivation of microbes and will have a role during this century in the prevention of many diseases. New fields of biotechnology applications and the procedures of inactivation of microorganisms have been discovered by using non-thermal plasmas. Recently, the field of glow discharge plasma in atmospheric pressure has rapidly expanded by using the application of atmospheric pressure plasma jet (APPJ), leading to the evaluation of the roles in the disinfection of bacteria of air plasmas at atmospheric pressure [1].

Microbial disinfection is important in many fields, especially in biological and medical fields. Disinfection is based on physical, microbiological, and chemical processes to eliminate microorganisms. Sterilization by gamma rays, chemicals such as ethylene oxide (ETO), autoclaves, and ovens are traditional methods of sterilization, all of them not comparable with treatment by new techniques, such as cold plasma, because plasma technology involves a complete disinfection and sterilization process in a very low exposure time for contaminated surfaces. However, traditional methods have their advantages as well as disadvantages [2].

The new fashion is to cover any contaminated surface with antimicrobial nanoparticles through spraying by APPJ without changing the surface’s shape, color, durability, or special fabric characteristics, but on the condition that antimicrobial materials are harmless, and are not affected by factors like humidity and changing weather [3].

Generated reactive hydroxyl and super oxygen radicals are responsible for cell damage through multiple stages, ending in complete cellular DNA degradation. It has been suggested that the antimicrobial effect of TiO_2_ on coated surfaces is effective against any type of microorganism, such as bacteria, viruses, and fungi [4].

The low gas temperatures at low pressures under vacuum, and atmospheric pressure glow discharge in air, opened new techniques and new fields of biotechnology applications, such as the effects of plasma on different bacterial genera [5]. Many papers deal with the evaluation of ultraviolet and reactive species due to high temperatures, and in the disinfection processes by the atmospheric pressure of plasma generated in the air [6]. A description of plasma interaction with microorganisms has been given [7], and all the required diagnostic techniques to deal with the atmospheric pressure of plasma generated in the air were also described [8]. Furthermore, roles for the research capability for biological/environmental applications have been studied using cold plasmas [9,10], taking into account that the investigated effects of non-thermal room temperature atmospheric pressure plasma plumes on microorganisms for biomedical applications [11].

From both electrical and biological outlooks, many of the active particles do not have a long lifetime in the environment. They are easily decayed or reacted with other components. Some active particles only have a life of milliseconds and even microseconds, and the longer treatment distance causes a longer time between the generation of active particles and their interaction with the bacteria [12].

The application of cold plasma by APPJ for the disinfection of microbes is shown to be safe. Biological safety is demonstrated through a series of differential skin toxicity trials on human and live mouse skin tissues [13].

The inactivation of microbial pathogens, such as viruses, fungi, and bacteria, and degrading toxins accumulated on any surface, such as agricultural products, to refine crop yields and food preservation, medical surgical tools for sterilizing therapeutic and dental devices, and disinfection and sterilization processes, all are considered in the current practical applications of APPJ technology [14].

A large range of environmental applications of cold plasma by APPJ has also been utilized, especially for the inactivation of microorganisms, the degradation of toxins, water purification, and remediation, as well as the treatment of exhaust gases. In future perspectives, using APPJ demonstrates a new opportunity for the scientific, engineering, and medical communities to prevent and treat coronavirus disease 2019 (COVID-19), which reflects one of the fashionable targets for recent studies [15].

In the present work, we suggest examining the inactivation characteristics of some microbes with a very short exposure time application, using a plume of cold plasma emerging from atmospheric pressure plasma jet (APPJ) to cause the inactivation of microbes attacking surfaces. Furthermore, many parameters affecting the inactivation of the microorganisms attached to the surface will be discussed by studying the survival curves using dry argon (0% O_2_) APPJ discharges and different wet argon discharges with admixtures with O_2_/Ar ratios ranging from 0.25% to 1.5%.Therefore, the axial distance between the jet and the sample was varied, and different powers of the jet and different applied voltages were used. The combined effects of direct and indirect exposure using a mesh controller, magnetic field strength, and membrane damage mechanism by APPJ will be discussed. Producing an inactivation process using non-thermal APPJ has been suggested for optimizing alteration parameters to achieve efficient protection from ultraviolet and electromagnetic irradiation.

## 2. Experimental Setup and Procedures

**Figure 1:**
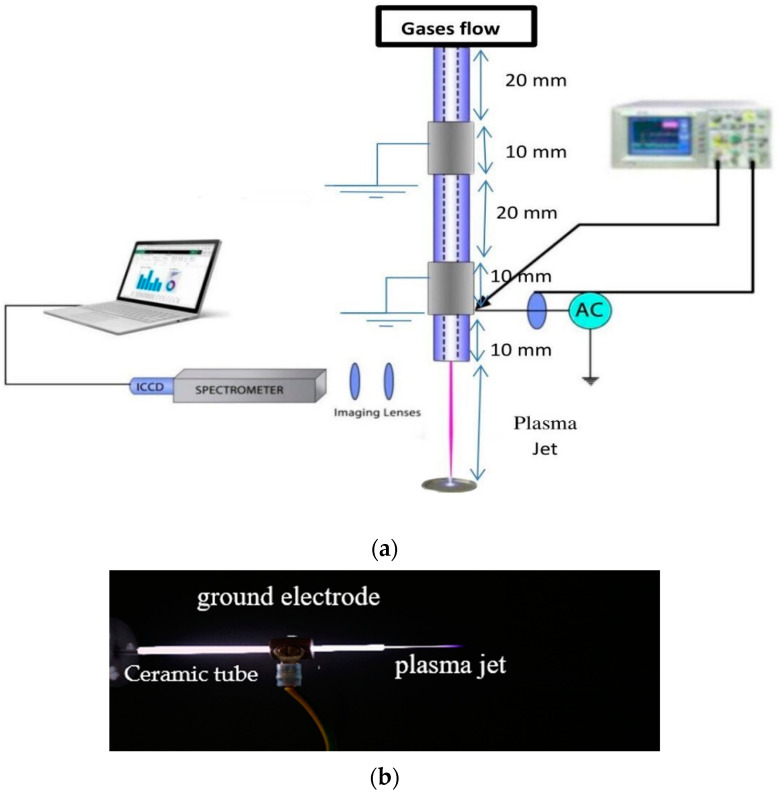
(**a**) Schematic diagram of atmospheric pressure plasma jet (APPJ) experimental setup; (**b**) typical example of the ground electrode as a calibration scale used in calculating plasma jet width and length.

The experimental set-up for the investigation of (non-thermal) atmospheric pressure plasma jet (APPJ) using different gases is shown in Figure 1a. It consists of a cylindrical ceramic tube with a length of 70 mm and a diameter of 2 mm, and two outer isolated copper electrodes (hollow cylindrical shape) around the ceramic tube. One electrode is powered at 60 kHz by a 15 W AC high-voltage source with a voltage ranging from 2.5 to 25 kV, and the other electrode is grounded at 20 mm from the high-voltage electrode and 40 mm away from the nozzle [16]. Moreover, the inner capillary represents the third electrode, attached at the end by the nozzle with a 1.5 mm diameter [17,18].

The length and the width of fast-repeated images of non-thermal plasma for emerging jet afterglow can be correctly measured relative to the dimensions of the ground electrode as a calibration scale, as shown in Figure 1b, the fast-repeated photo images are taken by a digital Canon camera (Ota City, Tokyo, Japan) with an exposure time of 40 ms. The calibrated length and width of the grounded electrode (10 mm and 3 mm, respectively) were taken as a reference for measuring the length and the width of the emerging jet using the Image J software (Java-based image processing program version 1.52v, Bethesda, MD, USA).

An avaspec-2048 spectrometer (Louisville, CO, USA) with CCD detector (Charge Coupled Device) was used to measure the axial distribution emission spectra of the APPJ, from the nozzle to a distance from the nozzle. The spectral range of the spectrometer was from 200 to 1100 nm and the spectral resolution was 1.4 nm, where the light emitted from the APPJ was collected by a lens at the end of a fiber optic cable.

Different gas admixtures were used, such as a mixture of argon with oxygen at different ratios, where the mass flow rate of each gas was controlled, and the gases flowed into a ceramic tube and the APPJ tip distance was fixed at an axial distance separation equal to 17.5 mm. Furthermore, the average plasma gas temperature was measured by a fluoroptic thermometer (Model No.604, Luxtron Corporation, Santa Clara, CA, USA).

The applied voltage U (kV) and discharge current I (mA) of the jet were measured with a Tektronix digital oscilloscope equipped with a high-voltage probe and a current probe.

Waveform signals of APPJ discharge were recorded by a 350 MHz digital oscilloscope, where the applied voltage was measured using a Tektronix P6015A (Beaverton, OR, USA) high-voltage probe, and the discharge current was observed by measuring the voltage over 33 kΩ.

Thermal, electrical, spectroscopical, and photographical characteristics of plasmas generated by APPJ were obtained. The APPJ system creates discharges using dry argon, i.e., pure argon with 0% O_2_, and different wet argon discharges, i.e., 1 slm argon admixture with oxygen ratios of 0.25, 0.50, 0.75, and1.5% O_2_, equivalent to 2.5, 5, 10, 15 mslm.

**Figure 2:**
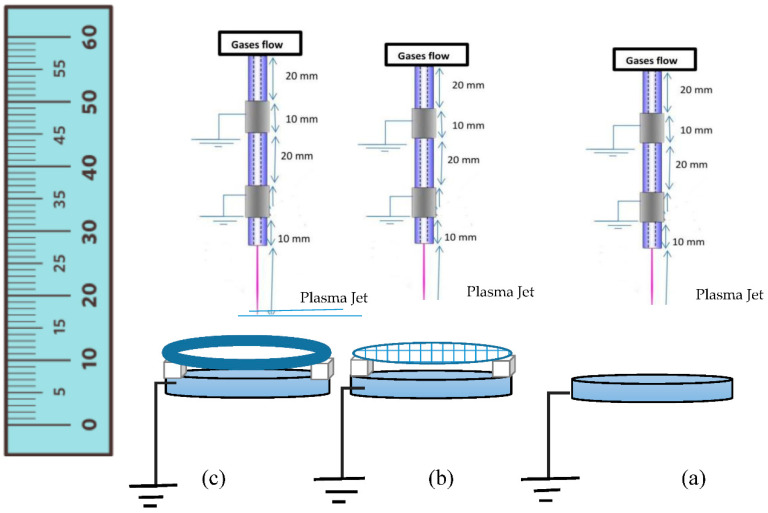
Schematic diagram of APPJ experimental set-up for the inactivation process of a bacterial sample in a Petri dish, for different configurations: (**a**) direct exposure, (**b**) indirect exposure using a mesh controller, and (**c**) indirect exposure using a hollow permanent magnet.

Figure 2 shows a schematic diagram of the APPJ experimental setup for the inactivation process of bacterial colony samples in a Petri dish, showing the plasma plume emerging from APPJ and contacting the samples for different configurations, such as direct exposure (Figure 2a), indirect exposure using a mesh controller (Figure 2b), and indirect exposure using a hollow permanent magnet (Figure 2c).

### 2.1. Microorganism Sample Preparation and Theory

The bacterial colony sample preparation was carried out as follows: a viable suspension of the bacterium was spread-plated onto a series of Petri dishes, with 60 mm diameter and 13 mm height, containing nutrient agar. The counting results are expressed as the bacterial concentration, each containing about 10^9^ colony-forming units per milliliter (CFU/mL) of Escherichia coli (*E. coli*) [17,18]. Five plates were exposed to dry and wet argon discharges, using a dry argon discharge (0% O_2_) and different wet argon discharge admixtures with oxygen ratios (0.25, 0.50, 1, and 1.5% O_2_) at different exposure durations. Furthermore, after exposure, the Petri dishes were incubated overnight at 37 °C for 24 h, and another five plates were kept as controls, not exposed to the previous conditions.

The bacterial colony sample inactivation theory can be discussed using the survival curves of living microbes studied after and before treatment of the microbes attached to any surface by APPJ [19,20]. The inactivation process of bacteria increased as the exposure time increased, and the sterilization efficiency is given by the logarithmic reduction value (R) using the following equation:

(1) $$\mathrm{Effect}\;\mathrm{of}\;\mathrm{Sterilization}\left(R\right)={\mathrm{Log}}_{10}\left[\frac{\;\mathrm{Population}\;\mathrm{before}\;\mathrm{treatment}\;N_{0}\;}{\mathrm{Population}\;\mathrm{after}\;\mathrm{treatment}\;N}\right]$$

where N_0_ and N represent the bacterial concentration before and after the plasma treatment with the unit of CFU/mL. The inactivation rate η can be determined from the following equation:

(2) $$\eta =\left[\frac{\;N_{0}\;-\;N}{\;N}\right]\times 100\%$$

The death value (D-value) represents the time necessary to destroy the initial microorganisms by 90% and can be expressed by the following equation:

(3) D−value= t [LogNtN0]

where t represents the time to destroy 90% of the initial population, and N_t_ is the total cell concentration after time {t} from the treatment.

### 2.2. Indirect Exposure Controls

An earthed aluminum mesh covered the Petri dish containing the bacterial colony samples was used to indirectly investigate the exposure to the cold APPJ plasma, i.e., the contribution of reactive species and charged particles to realize the disinfection efficiency. The mesh acts as an electrode for the APPJ with a mesh hole width of 3 mm, and several holes per inch, 8 holes/inch. The gap between the mesh and the Petri dish is a very important factor, 5 mm above the Petri dish, where the plasma contacts it indirectly through the mesh.

**Figure 3:**
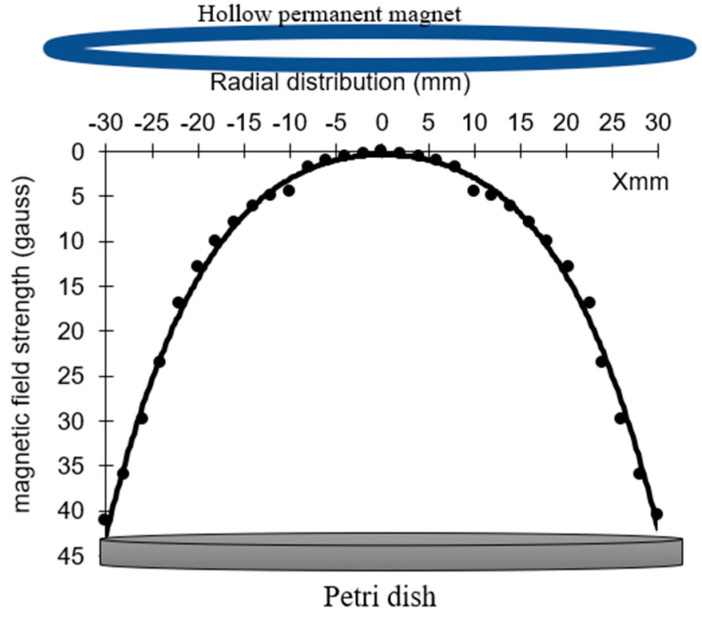
Radial distribution (mm) of the magnetic field strength (gauss) of the hollow magnet, from edge to edge of the Petri dish.

An earthed hollow circular permanent magnet with a diameter of 60 mm is used to produce the magnetic field strength (B). The magnet is placed over the Petri dish with an axial distance separation of 5 mm. Figure 3 shows the radial distribution of the magnetic field strength (B) from edge to edge of the Petri dish position at an axial distance of 5 mm. (B) has a maximum of 45 gauss at the Petri dish edges, and minimum of 2 gauss at its center, as measured with an LCD gaussmeter WT10A surface magnetic field tester, with a portable metal probe moving along the x-axis.

## 3. Results and Discussion

### 3.1. The Discharge Wettability Effect on Voltage–Current Signals

**Figure 4:**
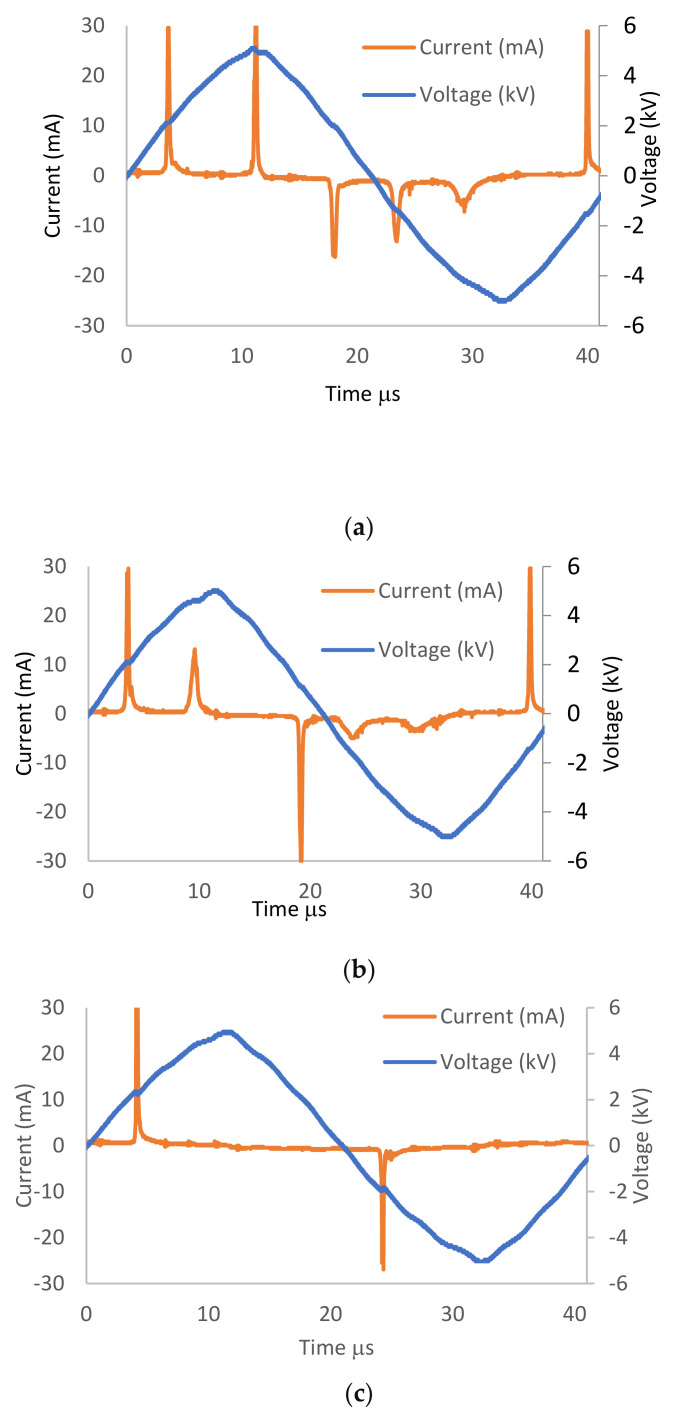
Voltage and current waveforms for (**a**) dry argon discharge at 1 slm, (**b**) wet argon discharge admixture with 0.5% O_2_, and (**c**) wet argon discharge admixture with 1.5% O_2_.

Figure 4a–d shows the influence of the argon–oxygen admixture with different oxygen ratios (0, 0.25, 0.50, and 1.5%) and an argon flow rate of 1 slm on the stability of voltage–current waveform signals of APPJ.

The applied voltage is sinusoidal for all waveforms, with 11.2 kV as peak-to-peak voltage, while the discharge current (I mA) consists of the displacement current [21]. A number of short peaks represent a few pulses of current, appearing at every half cycle, and these peaks appear because of the glow-like discharge when the plasma is formed [22,23]. For increasing O_2_ ratios, the number of peaks decreased due to the reduction of the sheath around the jet.

**Figure 5:**
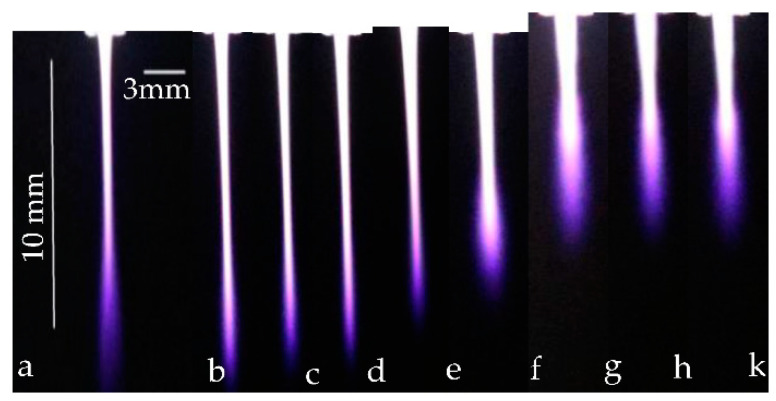
Photo-imaging of: (**a**) APPJ discharge using the ground electrode as a calibration scale used in calculating plasma jet width and length, (**b**) dry argon discharge, (**c**–**g**) wet argon with oxygen ratio from 0.25% to 1.5%, (**h**,**k**) indirect exposure using mesh and magnet controllers with 1.5% O_2_.

Figure 5a represents the photo-imaging of APPJ discharge, taken with a Canon digital camera in a darkened laboratory using the ground electrode as a calibration scale used in calculating jet width and length (10 mm and 3 mm, respectively). Figure 5b–g represents photo-imaging of direct exposure, dry and wet argon discharges with an oxygen ratio from 0% to 1.5%, and Figure 5h,k represents photo-imaging of indirect exposure wet discharges with 1.5% O_2_ using mesh and magnet controllers.

### 3.2. The Discharge Wettability Effect on Measured Parameters

**Figure 6:**
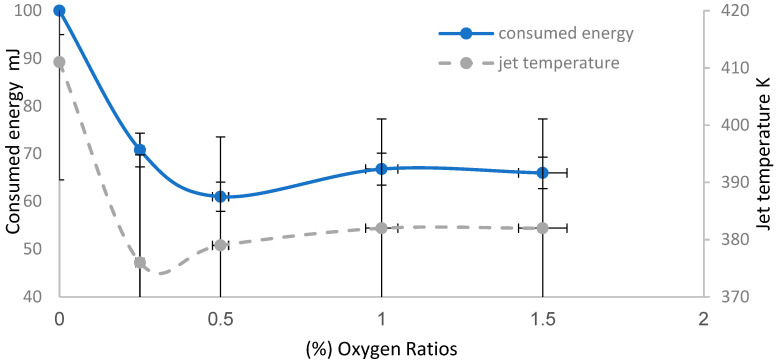
Discharge wettability effect on the consumed energy injected into the gas, and the jet temperatures at an axial distance equivalent to 17.5 mm using 1 slm of argon admixture with oxygen percentages from 0% to 1.5%.

Figure 6 shows the influence of discharge wettability effect at an axial distance equivalent to 17.5 mm using an argon admixture with oxygen ratios from 0% to 1.5%, on the consumed energy injected into the gas, and the jet temperatures. Using Voltage-Current waveform signals of the APPJ discharge, the discharge plasma power of the emerging jet can be computed, as in the following equation:

(4) $$P=\frac{1}{T}\int_{t}^{T}V\left(t\right)\;I\left(t\right)\;\mathrm{dt}$$

where P is the average power of the discharge (Watt), *T* is the period of the discharge (s^−1^), I(t) is the instantaneous current represented by the discharge current (mA), and V(t) is the instantaneous voltage represented by the peak-to-peak of the applied voltage (kV), obtained from the V-I waveform [24].

The values of discharge power decrease from 2.5 W at 0% O_2_ to 1.65 W at 1.5% [25]. The consumed energy can be estimated by dividing the discharge plasma power values by a frequency value tuned at 25 kHz (obtained from V-I waveform).

Figure 6 shows that as the oxygen ratios (wettability) increase, the consumed energies decrease from 100 mJ at 0% O_2_, to 66 mJ at 1.5%. It also shows that the temperature begins to decrease from 411 K and then reaches stable values at 382 K. This might be because as the oxygen ratio increases, inelastic collisions among particles increase, leading to losses in the discharge process, lower consumed energy, dissipated power processes, and lower jet temperatures [26,27]. This is useful to accelerate the disinfection process, since high thermal energy is transferred to the sample in the Petri dish due to the high thermal conductivity of oxygen and large inelastic cross-section of the mixed gases [28].

### 3.3. The Discharge Wettability Effect on the Dimensions

The width of the jet is an active and interesting parameter that depends on plasma jet length, and applied flow rates of argon and oxygen. The width is a guideline for the maximum area of irradiance emerging from the jet and reaching the exposed sample.

**Figure 7:**
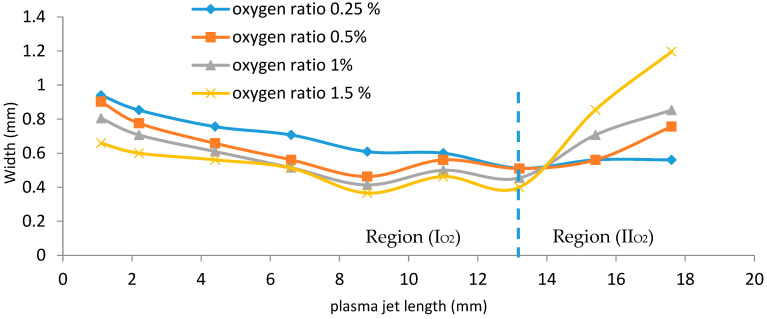
The relation between the plasma jet length and the width of the jet at different applied Figure. 0.5%, the length of the plasma jet ranging from 13.2 mm to 17.5 mm gives the biggest widths of the jet.

Figure 7 shows that as the wettability of oxygen increases, from 0.25%, 0.5%, and 1% to 1.5% in the admixture with 1 slm of Ar, there are two behaviors that are observed:

(a) Region (I_O2_) in Figure 7: as the wettability of oxygen increases, the width dramatically decreases axially to minimum values in the range of 0.512 to 0.4 mm at a plasma jet length of 13.2 mm (represented by the region to the left-hand side of the vertical dashed line).
(b) Region (II_O2_) in Figure 7: as the wettability of oxygen increases, the width begins to increase until reaching a wider scale, in the range of 0.561 to 1.196 mm at a plasma jet length of 17.6 mm (represented by the region to the right-hand side of the vertical dashed line).

This means that, for region (II_O2_), as the admixture values of oxygen flow rates increase, the width dimensions of the plasma jet increase, and the axial length dimensions of the jet are elongated.

**Figure 8:**
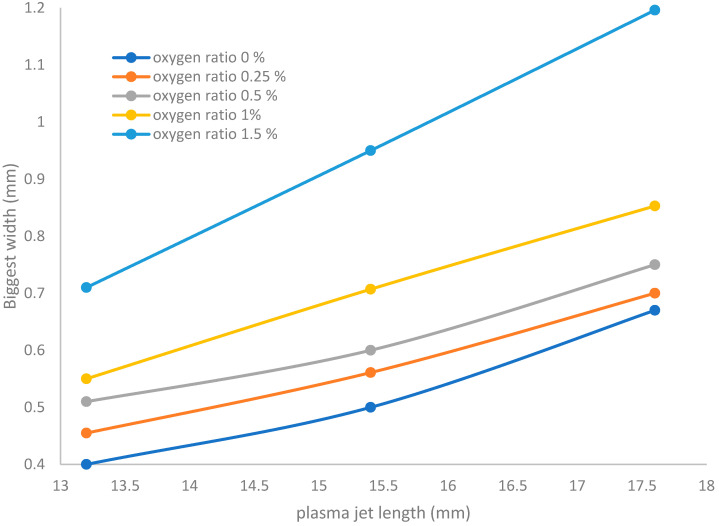
Relation between the plasma jet length and biggest width of the jet at different applied wettabilities of oxygen.

Figure 8 shows that for the discharge wettability ranging from 0% to 1.5%, the widths of the jet ranging from 0.4 to 0.71 mm at an equivalent jet length 13.2 mm and ranging from 0.67 to 1.19 mm at an equivalent jet length 18.3 mm give the maximum area for irradiance covering the exposed sample, whereas when the admixture values of oxygen flow rates increase, the width dimensions of the plasma jet increase until reaching a wider scale.

**Figure 9:**
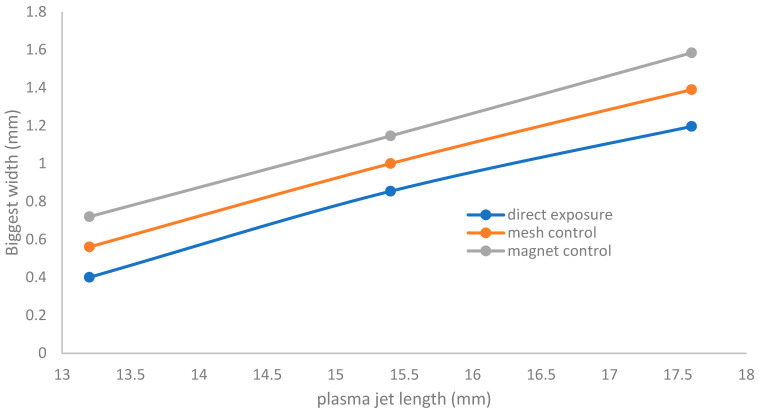
The relation between the plasma jet length and biggest width of the jet at a discharge wettability ratio of 1.5% O_2_ for direct exposure and indirect exposure (mesh controller and magnet controller).

Figure 9 shows the relation between the plasma jet length and biggest width of the jet at a discharge wettability ratio of 1.5% O_2_ for direct exposure and indirect exposure (mesh controller and magnet controller) for an axial jet length ranging from 13.2 to 18.3 mm. It is concluded that the jet width (J_w_) increases more for indirect exposure than for direct exposure, as in the following equation:

(J_w_)_magnetic field controller_ > (J_w_)_mesh controller_ > (J_w_)_direct exposure_ (5)

The discharge wettability ratio from 0.25% to 1.5% gives the biggest widths of the jet because the flow mode was laminar. In contrast, if the oxygen wetness increases more than 1.5%, the width dimensions not matching the results may be because the flow mode changed from laminar to turbulent flow. Furthermore, if the oxygen wettability was more than 1.5%, a sheath was created around the tube, the mesh, and the magnet. Moreover, some reasons for the largest values of jet widths will be shown and discussed in Section 3.7.1 and Section 3.7.2, especially when using mesh and magnet controllers.

### 3.4. The Discharge Wettability Effect on the Optical Emission Spectroscopy

The optical emission spectroscopy (OES) data of APPJ for the 1 slm argon discharge wettability with 0% 0.25%, and 1.5% O_2_, at the center of the jet vertical axis using fiber optics, was investigated. The relationship between the intensity of emission spectra (IES) is plotted versus the wavelength ranging from 200 to 900 nm, with an axial length of 17.5 mm from the nozzle.

**Figure 10:**
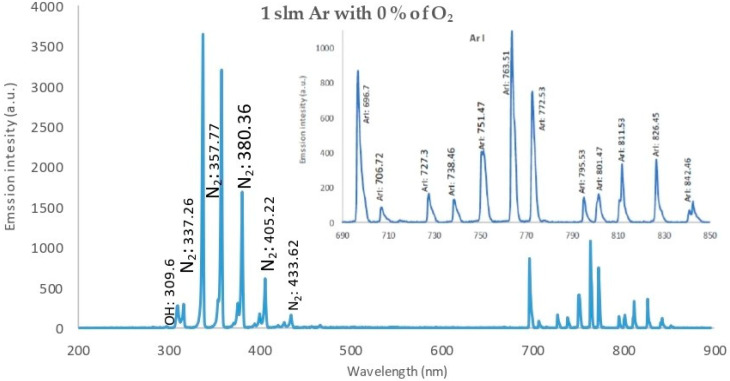
Relationship between intensity of emission spectra of APPJ, for the discharge wettability at 0% O_2_, and the wavelength, ranging from 200 to 900 nm, with an axial length of 17.5 mm from the nozzle.

Figure 10 displays the OES of 1 slm of pure argon (0% O_2_ wettability) discharge, a high emission intensity with a strong line for the hydroxide band (OH) at 309.6 nm ((A^2^∑^+^-X^2^∏) transition), beside the nitrogen band (N_2_): {337.26, 357.77, 380.36 nm} ((C^3^Π_u_-B^3^Π_g_) transition). The Ar lines are as follows: ArI: {696.7, 727.3, 738.46, 751.47, 763.76, 772.53, 795.53, 801.47, 811.53, 826.45, and 842.46 nm} (3*s*^2^3*p*^5^(^2^P°_3/2_)4*p* transition).

**Figure 11:**
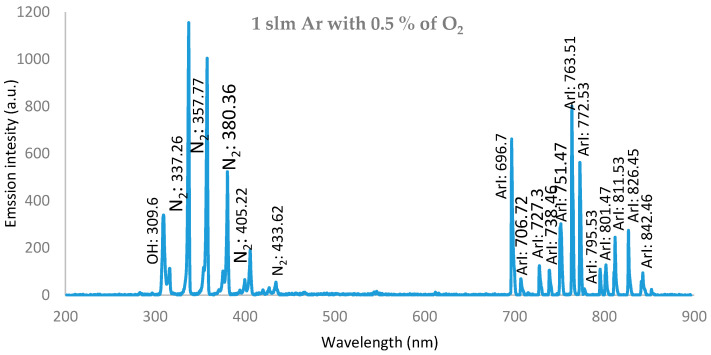
Relationship between intensity of emission spectra of APPJ, for the discharge wettability at 0.5% O_2_, and the wavelength, ranging from 200 to 900 nm, with an axial length of 17.5 mm from the nozzle.

Figure 11 shows the following OES data for the O_2_/Ar wet discharge with 0.25% O_2_, and a moderate emission intensity with Ar lines appeared, as for the case of pure argon discharge presented in Figure 10, beside oxygen (O) radical lines at: {777.84 and 843.8 nm} (3*s*^2^3*p*^5^(^2^P°_3/2_)4*s* transition).

**Figure 12:**
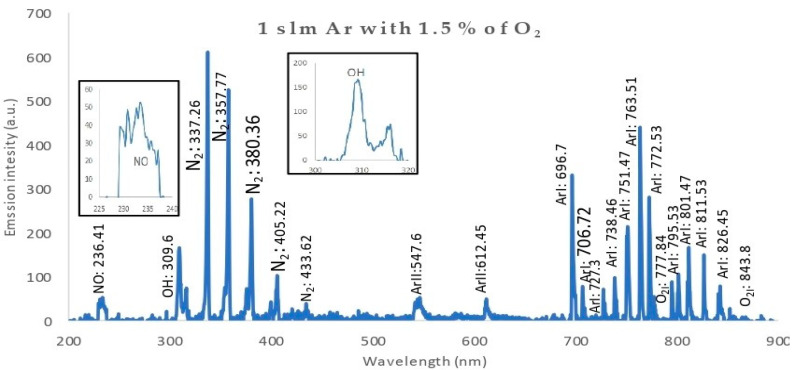
Relationship between intensity of emission spectra of APPJ, for the discharge wettability at 1.5% O_2_, and the wavelength, ranging from 200 to 900 nm, with an axial length of 17.5 mm from the nozzle.

Figure 12 shows a lower emission spectrum intensity for the discharge wettability at 1.5% O_2_: a nitric oxide (NO) band at 236.41 nm ((A^2^∑^+^-X^2^Π)transition), a hydroxide band (OH) at 309.6 nm ((A^2^∑^+^-X^2^∏) transition), N_2_ bands at {337.26, 357.77, 380.36, 405.22, 433.62 nm} ((C^3^Π_u_-B^3^Π_g_) transition), along with the plasma jet due to the mixing of the wet argon with the surrounding ambient air [29]. Furthermore, there are Ar lines: ArII: {547.6 and 612.45nm} and ArI: {696.7, 706.72, 727.3, 738.46, 751.47, 763.76, 772.53, 795.53, 801.47, 811.53, 826.45 nm} (3*s*^2^3*p*^5^(^2^P°_3/2_)4*p* transition), showing that the discharge was dominated by electron impact ionization [30]. Oxygen (O) radical lines occur at {777.84 and 843.8 nm} (3*s*^2^3*p*^5^(^2^P°_3/2_)4*s* transition).

Generally, the discharge wettability effect on OES showed that:

- IES decreases as wettability increases.
- The presence of OH, O, and N_2_ bands and lines are attributed to the interaction of ambient air with excited argon species, as well as high-energy electrons in the plasma.
- The OH^•^ and O^•^ radical emissions were due to impurities in the gas or due to the entry of air into the discharge zone [31].
- Since the reactive species such as O, OH, and NO are the most effective agents in biomedical applications, the plume interacts with ambient gases and molecules. Atomic (O) and (OH) radicals play important roles in the bacterial inactivation, especially in wound healing applications. Furthermore, reactive oxygen and nitrogen species (ROS and RNS) can accelerate the inactivation process by diffusing into cells [32].
- The oxygen/argon admixture discharge shows the same trend and behavior as for pure argon discharge but with lower values of intensity, which may be due to the decrease in electron density and temperature as the oxygen wettability increases, which may be due to higher dissociation of the oxygen molecules in the discharge processes [33].

### 3.5. The Discharge Wettability Effect on the Inactivation Process

Table 1 shows photos of Petri dishes, with Table 1A representing the control Petri dish without any exposure, containing about 10^9^ CFU/mL of *E. coli*, and Table 1B–G representing Petri dishes exposed to the plasma jet, with an applied voltage of 11.2 kV and wettability of 1.5% O_2_, using three different methods: direct exposure in Table 1B–D, grounded mesh controller in Table 1E–G, and grounded magnetic field controller in Table 1H,K,L. The inactivation region appears as a hole in the etched surface of the *E. coli* culture and becomes a wider sterilized semi-circle region in the center, which represents the growth of the inactivation process.

**Figure 13:**
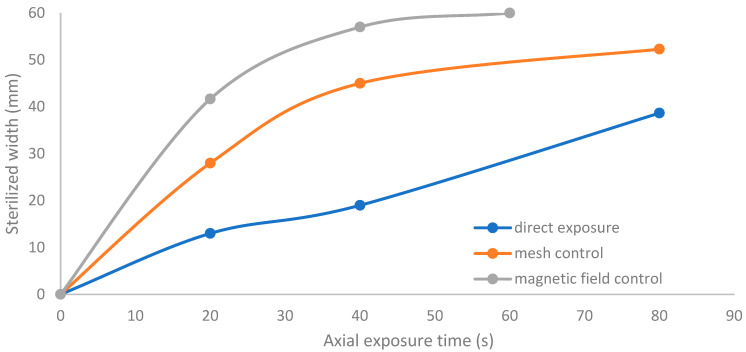
Increase in sterilized width (σ_w_) with exposure time for the discharge wettability at 1.5% O_2_ using the three different exposures: direct exposure, grounded mesh controller, and grounded magnetic field controller.

Figure 13 shows the growth of the sterilized width (σ_w_) for the discharge wettability at 1.5% O_2_ using the three different exposures: direct exposure, grounded mesh controller, and grounded magnetic field controller. σ_w_ increases as the exposure time increases, as in the following equation:

(σ_w_)_magnetic field controller_ > (σ_w_)_mesh controller_ > (σ_w_)_direct exposure_ (6)

The diameter of the Petri dish (60 mm) is considered as a reference measurement of the etched hole widths.

### 3.6. The Discharge Wettability Effect on the Survival Curves

The inactivation process of *E. coli* samples was studied by survival curves, plotting the logarithm of colony-forming units per milliliter (CFU/mL) (representing the bacteria logarithmic reduction), versus the exposure time of plasma emerging from APPJ [34,35] using dry argon discharges (0% O_2_) and different wet argon ratio discharges ranging from 0.25% to 1.5% O_2_, with the samples detected at 17.5 mm as an axial location from the jet nozzle.

**Figure 14:**
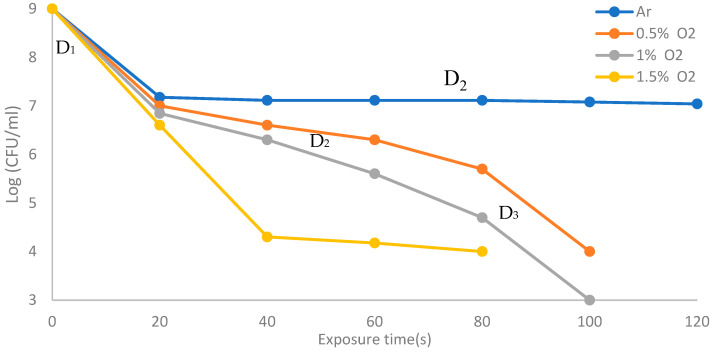
The survival curves using direct exposure for the discharge wettability ranging from 0% to 1.5% O_2_.

Figure 14 shows the survival curves using direct exposure for the discharge wettability ranging from 0% to 1.5% O_2_. For dry argon with 0% O_2_, living bacteria can be almost completely inactivated within 120 s with two phases (D_1_ and D_2_). The inactivation rate is similar within 100 s for wet argon with 0.5% O_2_ and 1% O_2_ but with three phases (D_1_, D_2_, and D_3_), and reaches 80 s for wet argon with 1.5% O_2_ with two phases. The experimental results show that as the wettability of oxygen ratios increases under the discharge, the inactivation rate increases with increasing exposure time.

Phases 1 and 2 represent the limited penetration depth of plasma jet discharge due to radicals, electrons, and positive ions, where the destruction of isolated viable cells depends on the erosion rate of the various materials (coats, debris, dead cells) covering still viable cells [36]. Phase 3 deals with residual living cells that were not sufficiently inactivated during phases 1 and 2.

A variety of active particles is created depending on the plasma ionization degree and determines the inactivation effect. Dry argon has the poorest inactivation effect since the inert Ar gas is very difficult to ionize and thus to produce enough active species for the inactivation. As the wettability increases, the atomic O radicals, OH radicals, ROS, and RNS increase, producing a decrease in the exposure time and hence accelerate the inactivation process [37,38]. In contrast, a lower wettability gives a lower concentration of active particles, and a smaller etching effect on bacteria.

### 3.7. Indirect Discharge Effect

**Figure 15:**
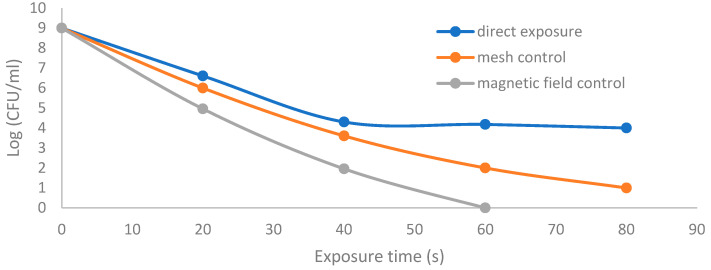
The survival curves for the discharge wettability at 1.5% O_2_ using the three different exposures: direct exposure, grounded mesh controller, and grounded magnetic field controller.

Figure 15 shows the CFU logarithmic reduction for the discharge wettability at 1.5% O_2_ using the three different exposures: direct exposure, grounded mesh controller, and grounded magnetic field controller. The experimental results for both controllers show a shorter treatment period, faster inactivation effect and, furthermore, with one phase, D_1_, the magnetic field controller gives a faster inactivation than the mesh controller.

**Figure 16:**
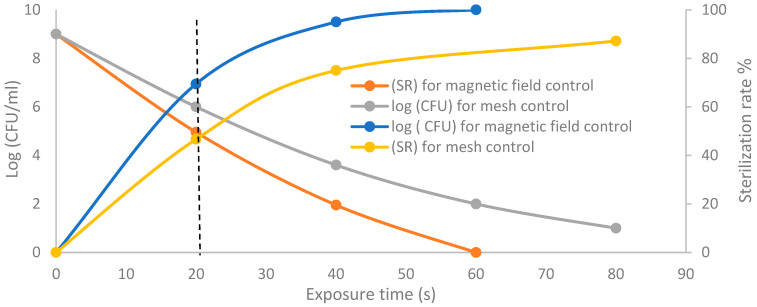
Colony-forming unit (CFU) logarithmic reduction and sterilization rate (%) versus exposure time for the discharge wettability at 1.5% O_2_ using grounded mesh controller and grounded magnetic field controller.

Moreover, Figure 16 shows the CFU logarithmic reduction and sterilization rate (SR) (%) at different axial exposure durations, for the discharge wettability at 1.5% O_2_, using the grounded mesh controller and grounded magnetic field controller. The results of plasma jet inactivation are mainly divided into two periods of 0 to 20 s and 20 s to 80 s.

The first period exhibits a moderate inactivation process within 20 s before the dotted line. For the magnetic field controller, the CFU logarithmic reduction is 4.9 with a sterilization rate (SR) reaching 69.4%, while for mesh controller, the CFU logarithmic reduction is 6 with an SR reaching 46.6%.

The second period after the dotted line exhibits a rapid inactivation process within 80 s. For the mesh controller, the CFU logarithmic reduction is 1.2 with an SR reaching 87.1%, while for the magnetic field controller, the CFU logarithmic reduction is 0.2 with an SR reaching 99% within 60 s. The photos of Table 1H,K,L represent the grounded magnetic field controller using a hollow magnet with a higher etching rate and better inactivation effect than in the photos of Table 1E–G, representing the grounded mesh controller. This may be attributed to important factors such as the mesh controller and magnetic field controller, as follows.

#### 3.7.1. Mesh Controller

Mesh parameters are very important factors for the indirect contact of the plasma and Petri dish [39,40,41], as shown in the recent article in which a grounded aluminum mesh above a Petri dish acts as an electrode for the APPJ. The mesh hole width (3 mm) allows a high rate of heat transfer from the gap (5 mm), between the wire mesh and the culture media in the Petri dish, despite energy losses due to scattering from the wires and the largest sheath length (λ) around the mesh wires. This may be due to the low electron-neutral particle collision frequency λ_e−n_ and the large mean free path λ_e−n_. Hence, the loss of electron energy is low, and the rate of plasma loss by diffusion coefficient D is given in the following equation [42]:

(7) $$D=\;\frac{\mathrm{KT}}{{m\;\nu }_{e-n}}$$

The number of holes per inch (8 holes/inch) is enough to ionize the gas in the surrounding area of the sharp edges of the mesh holes, attributable to the Penning effect, and leads to the production of a higher local electric field strength for Al mesh, and to a high heating effect for the disinfection process [43].

The Al mesh accelerates the charged particles due to the increase in the electric field between the mesh and electrodes of the APPJ, and enhances the interaction between charged particles and other particles to generate more reactive species, such as OH and O, showing the important role of charged particles in increasing the killing area of the inactivation process [44] by using a mesh controller in comparison with direct exposure, although less than with a magnetic field controller.

#### 3.7.2. Magnetic Field Controller

The mapping of the magnetic field lines in the discharge gap between the magnet and the Petri dish is discussed briefly in Section 2.2, investigating the important role of the magnetic field controller. Due to the hollow magnet, the charged particles in the plasma jet emerging from the APPJ nozzle gyrate around the magnetic field lines in circular orbits with Larmor radius r_L_ and the jet velocity v as in the following equation [45]:

(8) $$r_{L(e,+)}=\frac{{\;m}_{\left(e\;,\;+\right)}{\;v}_{\left(e\;,\;+\right)}}{e\;B}$$

where B is the field strength, e is the electron charge, and m is the mass of the charged particles.

As B increases, v increases and r_Le_ decreases, and the jet penetrates the surface of the sample faster than for direct exposure. The velocity of the charged particles, due to the hollow magnetic field beside their velocity in the jet emerging from the nozzle, accelerates the sterilization process and decreases the exposure time to complete the process, especially at the edge of the Petri dish where high values of B and v occur. This is the main reason for the largest values of jet width, as shown in Section 3.3 and Figure 9.

Energy dissipation due to the discharge process decreases by using a hollow magnet because the diffusion coefficient in the presence of the magnetic field is dramatically decreased compared with direct exposure to the plasma jet [46], as in the following equation:

(9) $$D_{B}=\frac{D}{1+{\left(\;\frac{\mathrm{eB}\;\tau \;}{m}\right)}^{2}\;}$$

τ is the mean time between collisions, and D_B_ and D are the diffusion coefficients in the presence and absence of a magnetic field, respectively.

### 3.8. Membrane Damage Mechanism by APPJ

It is concluded from the previous sections that the inactivation of E. coli occurs using APPJ with dry and wet argon discharges, and with direct and indirect exposure. Plasma temperature from the jet, the distance between the jet and the sample membrane, and plasma exposure time are very important factors in the inactivation process. The membrane damage mechanism of *E. coli* by APPJ can occur by the following factors:

- The charged particles created by APPJ are collected on the surface of the cell membrane. These charges form an electrical force which can penetrate the tensile strength of the cell membrane, where the destruction of isolated viable cells depends on the erosion rate of the varied materials (coats, debris, dead cells) covering still viable cells. Atomic O and OH radicals play important roles in the bacterial inactivation, especially in wound healing applications [47].
- Reactive oxygen and nitrogen species (ROS and RNS) can accelerate the inactivation process by diffusing into cells. ROS and RNS increase, producing a decrease in the exposure time and hence an acceleration of the inactivation process since they target proteins, DNA, cell walls, and membranes, causing a leakage of the cell wall, and inducing DNA deterioration, which successively causes the inactivation of bacteria [48].
- An external magnetic field plays a major role in the process of bacterial inactivation as it accelerates the sterilization process and decreases the exposure time to complete the process, especially at the edge of the Petri dish where there are high values of magnetic field strength and charged velocities [49].

## 4. Conclusions

The main important measured parameters, such as discharge current, discharge applied voltage, discharge power, consumed energy injected into the gas, width of the jet, charged particle sputtering of the samples, axial distance between the APPJ nozzle and the sample, and exposure time for direct or indirect exposure, are all shown to be important factors determining the amount of heat transferred to the contaminated samples in the inactivation process.

The main idea behind direct or indirect exposure is discussed for a plasma plume emerging from APPJ in contact with microorganisms attached to skin or a sample from a wall or bathroom. All characteristics of the generated plume affecting the microbial disinfection process of *E. coli* bacteria attached to silk were investigated.

As the concentration of oxygen in argon increases, the wettability of the discharges increases, and charged particles and active radical species can reach the Petri dish and give a strong inactivation ability. The area of the inactivated region is much greater with a shorter treatment exposure time and a faster inactivation process by using a mesh controller and magnetic field controller.

The significance of the gap between the wire mesh or the hollow magnet and the culture media in the Petri dish for both indirect exposure methods deals with the culture media at the edges of the Petri dishes, accelerates the inactivation of microbes, increases the heat energy transfer to the colonies, inactivates media culture in corners and places that are difficult to reach, and quickly prevents virus transmission from urinals and toilets.

The directions for our future work include an experimental study to compare the disinfection process by inactivating bacteria using APPJ coupled with titanium dioxide, which is characterized by the sterilization and the elimination of organic matter and the self-cleansing of surfaces.

## Figures and Tables

**Table 1:** Photos of Petri dishes containing E. coli. (**A**) the control Petri dish and (**B**–**G**) Petri dishes exposed to the plasma jet, with an applied voltage of 11.2 kV at different exposure durations; using discharge wettability at 1.5% O_2_ represented by three different methods: direct exposure in (**B**–**D**), grounded mesh controller in (**E**–**G**), grounded magnetic field controller in (**H**,**K**,**L**).

		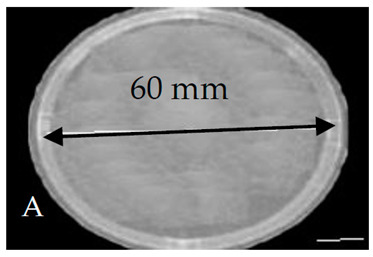	
Exposure time (s)	20 s	40 s	80 s
Direct exposure	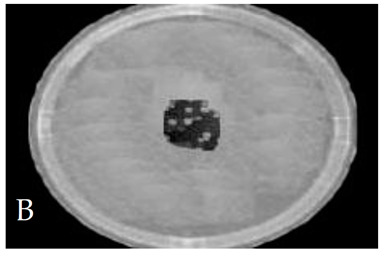	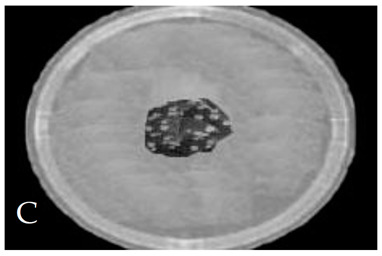	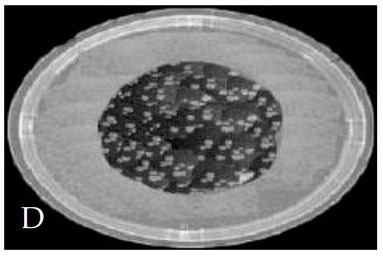
Indirect exposure (mesh controller)	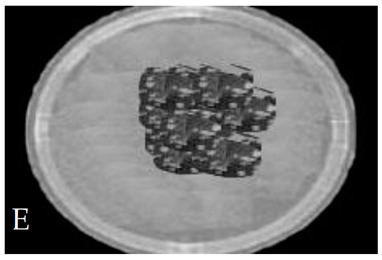	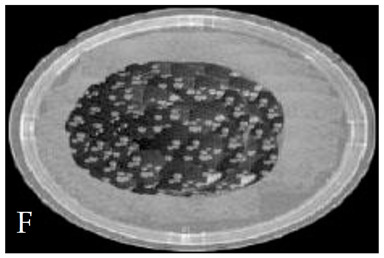	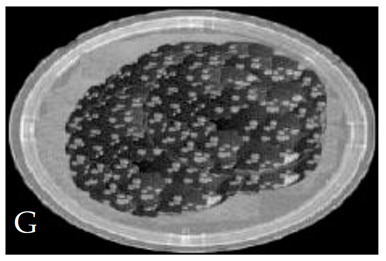
Indirect exposure (magnetic field controller)	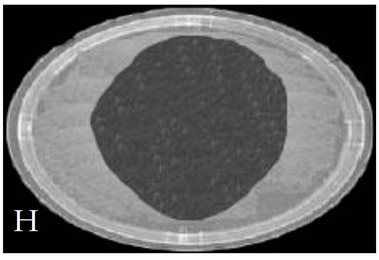	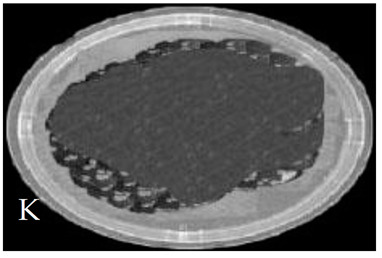	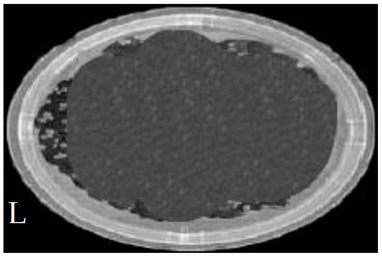

## Data Availability

Data is contained within the article.
